# Culturable Bacterial Endophytes From Sedimentary Humic Acid-Treated Plants

**DOI:** 10.3389/fpls.2020.00837

**Published:** 2020-06-19

**Authors:** David De Hita, Marta Fuentes, Angel M. Zamarreño, Yaiza Ruiz, Jose M. Garcia-Mina

**Affiliations:** ^1^Department of Environmental Biology, Biological and Agricultural Chemistry Group (BACh), University of Navarra, Pamplona, Spain; ^2^Centre Mondial de I’lnnovation (CMI) – Groupe Roullier, Saint-Malo, France

**Keywords:** endophyte, plant growth promotion, microbiota, humic, phosphate solubilizing bacteria, cucumber

## Abstract

The global decrease in soil fertility leads to a new agricultural scenario where eco-friendly solutions play an important role. The plant growth promotion through the use of microbes, especially endophytes and rhizosphere microbiota, has been proposed as a useful solution. Several studies have shown that humic substances are suitable vehicles for the inoculation of plant growth promoting bacteria, and that this combination has an enhanced effect on the stimulation of plant development. In this work, cucumber plants grown hydroponically have been pre-treated with a sedimentary humic acid (SHA) with known plant growth-enhancing effects, and culturable bacterial endophytes have been isolated from these plants. The hypothesis was that this pre-treatment with SHA could lead to the isolation of certain endophytic taxa whose proliferation within the plant could have been promoted as a result of the effects of the treatment with SHA, and that could eventually reinforce a potential synergistic effect of a combined application of those endophytic bacteria and SHA. The culturable endophytes that have been isolated from humic acid-treated cucumber plants have been identified as members of four main phyla: *Proteobacteria*, *Firmicutes*, *Actinobacteria*, and *Bacteroidetes*. Isolates were characterized according to the following plant growth-promoting traits: nitrogen fixation/scavenging, phosphate solubilization, siderophore production and plant hormone production. Most of the isolates were able to fix/scavenge nitrogen and to produce plant hormones (indole-3-acetic acid and several cytokinins), whereas few isolates were able to solubilize phosphate and/or produce siderophores. The most promising endophyte isolates for its use in futures investigations as plant growth-promoting bacterial inocula were *Pseudomonas* sp. strains (that showed all traits), *Sphingomonas* sp., *Stenotrophomonas* sp. strains, or some *Arthrobacter* sp. and *Microbacterium* sp. isolates.

## Introduction

In the last decades the human population has grown exponentially, reaching 7,600 million people in 2018, and as the Food and Agriculture Organization (FAO) has predicted, in 2050 the world population will be near to 10,000 million^1^. This fact involves an increasing pressure over global food production and the land surface dedicated to that purpose. However, ∼35% of the world surface is already dedicated to crop production, according to FAO database^2^, and increasing the crop land surface is not an ecologically valid solution, being in fact very controversial in most of the developing countries where population demands new eco-friendly politics. Only increasing crop yields appears as a possible solution to prevent food shortage in this future scenario, although the excessive use of NPK chemical fertilizers is already negatively affecting soil fertility, soil microbial activity, and may cause the pollution or/and eutrophication of water reservoirs (Torrent et al., 2007; Ulén et al., 2007; Youssef and Eissa, 2014).

Therefore, more rational, environmentally friendly, and efficient agricultural practices are needed. One approach is the use of biofertilizers containing living microorganisms (Dastager et al., 2010; Bhardwaj et al., 2014; Canellas et al., 2015; Pérez et al., 2016; Suhag, 2016; Dias et al., 2017). This strategy has recently gained relevance with the development of a new generation of gene sequencing techniques, which have allowed the assessment of microbe-plant relationships and the development of a new evolutionary model, the holobiontic theory (Rosenberg and Zilber-Rosenberg, 2016). This model proposes that microbiota would evolve over time to improve the fitness of the plant under changing environmental conditions such as drought, salinity, nutrient deficiency, or soil contamination (Murphy et al., 2015; Fidalgo et al., 2016; Soussi et al., 2016; Kumar et al., 2017).

Among the different kinds of biofertilizers, those including plant growth-promoting rhizobacteria (PGPR) are frequent (Mahaffee and Kloepper, 1997; Bhattacharyya and Jha, 2012; Sarathambal et al., 2015; Kumar et al., 2017; Gouda et al., 2018). The main effect of PGPR in plants is the improvement of both nutrient availability in the rhizosphere and the plant resistance to biotic/abiotic stresses (Gouda et al., 2018). However, the application of these microorganisms has several efficiency limitations when applied to the soil under field conditions due to the competition with native soil microbiota and their low survival rate. These facts cause the poor reproducibility of the agronomical results of PGPR-based treatments in field crops (Oliveira et al., 2006). Recent studies have shown that a promising approach to overcome all these limitations in efficiency might be the application of PGPR directly on the leaves (Canellas et al., 2013, 2015; Olivares et al., 2017).

In contrast with PGPR, the endophytic microbiota has been only recently explored as a potential source of beneficial microorganisms for improving plant growth (Brader et al., 2013; Sessitsch et al., 2019). Endophytes are those microorganisms inhabiting inner plant tissues (Hallmann et al., 1997). Their main source is the rhizosphere so that they share the same advantages as those of PGPR but showing special characteristics that may overcome some of the limitations associated with the use of PGPR even when applied to the leaves. Endophytes are well adapted to living within the plant, thus favoring in some way their efficacy as plant growth promoters (Reiter and Sessitsch, 2006; Hardoim et al., 2008; Compant et al., 2010). Indeed, endophytes have evolved to transmit themselves to the next plant generation through seed colonization (Johnston-Monje and Raizada, 2011; Truyens et al., 2015; Nelson, 2018). In fact, this trait would justify the use of PGP endophytes instead of PGPR inoculums: the evolutionary selection of endophytes and the capability of being inherited through plant generations provide them with high biocompatibility with plant tissues thus increasing their possibilities to help plants to grow under normal conditions or under stress conditions (López et al., 2018).

On the other hand, several studies have shown the compatibility and synergistic beneficial action on plant growth of PGPR and humic substances (HS) when applied together (Olivares et al., 2017 and references therein), the most studied combination being with diazotrophic endophytic bacteria, especially *Herbaspirillum* spp. HS are a specific fraction of soil organic matter that can be extracted using alkaline solutions (Stevenson, 1994) and have been proven to promote the plant development by increasing nutrient availability in soils and activating plant metabolism (Mora et al., 2010, 2013; Zandonadi et al., 2013; García et al., 2014; Olaetxea et al., 2018; de Hita et al., 2019; Zanin et al., 2019).

The aim of the present work was to isolate the culturable endophytic bacteria from plants pre-treated with HS. The HS used was a sedimentary humic acid (SHA) with a known plant growth-enhancing effect on cucumber plants (Aguirre et al., 2009; Mora et al., 2010, 2013; Olaetxea et al., 2015). To the date of the preparation of this manuscript, there were no published papers about how HS affect the endophytic microbial populations, with the exception of de Hita et al. (2018). In that preliminary study, the data showed that the application of SHA can modulate the relative abundances of some bacterial (i.e., *Actinobacteria*) and fungal endophytic communities in cucumber plants, based on cultured-independent techniques (metagenomics sequencing). In this context, our approach has been to pre-treat the plants with SHA, and to isolate the culturable endophytes, whose proliferation (or at least the proliferation of some of them) within the plant might have been helped by the application of SHA. The PGP traits of each isolate have then been tested. The ultimate goal of this work was to identify potentially promising endophytic PGP bacterial candidates isolated from plants pre-treated with SHA, with the hypothesis that they could also show a synergistic effect when applied as inocula in combination with SHA, based on the review by Olivares et al. (2017).

## Materials and Methods

### Plant Material and Growth Conditions

Cucumber seeds (*Cucumis sativus* L. var. Ashley) were sown in a bed of sterile perlite and wet filter paper, and placed in a germination chamber in darkness, at 25°C, and 75% relative humidity. One week later the seedlings were transferred to a hydroponic system in a growth chamber whose day/night conditions were: 16 h/9 h (irradiance of 250 μmol m^–2^ s^–1^), 25°C/21°C and 70%/75% relative humidity. The nutrient solution utilized was previously described in Mora et al. (2010) and Olaetxea et al. (2015), with minimum changes in the final concentration of Fe-EDDHA and MnSO_4_ (80 and 27.3 μM, respectively). After 10 days, plants were treated with a 100 mg L^–1^ C of a SHA obtained from leonardite as described in Mora et al. (2010) and characterized in Aguirre et al. (2009). The treatment was applied 2 h after the start of the diurnal period. Plants were harvested 7 days from the onset of the treatments.

### Plant Surface Sterilization and Bacterial Endophyte Isolation

SHA pre-treated cucumber plants were surface-sterilized prior to the isolation of bacterial endophytes. Firstly, three different cucumber plants were rinsed, separately, with autoclaved deionized water (dH_2_O) to wash the nutrient solution from the roots. Plant surfaces were afterward sterilized following a protocol based on the methods reported in Hardoim et al. (2012) and Reinhold-Hurek et al. (2015), with little modifications. Briefly, cucumber plants were rinsed with commercial bleach (<5% sodium hypochlorite) containing 0.1% Tween 20 for 3 min. The next steps consisted of three consecutive washes with autoclaved dH_2_O for 5 min each one and stirring in an orbital shaker. Finally, roots, stem, and leaves were separated with a sterile scalpel and frozen with liquid N_2_ for later use. To verify the surface sterilization, 1 mL of the last wash was plated and cultured on R2A agar medium. Plates were incubated at 27°C for 3 days. No growth was detected for any plant. All sterilization steps were carried out in a laminar flow cabinet in sterile conditions.

Plant organs were ground with sterile mortar and pestle using autoclaved peptone water (0.9 mL per tissue gram) to recover the microorganisms. The liquid was filtered through sterile gauze to eliminate plant debris. This filtrate was used (100 μL) for microbial culturing 10-fold serial dilutions in autoclaved peptone water (10^0^–10^–4^).

Microorganisms were isolated plating one milliliter from each dilution, by the pouring plate method, in a minimal medium (R2A agar) with the aim to favor the slow-growing bacteria from endosphere (Eevers et al., 2015, 2016). Plates were incubated for 7 days at 27°C. Morphologically single colonies from each plate were selected, picked, streaked, and re-streaked on new R2A agar plates to obtain axenic cultures of each isolate. Finally, each pure culture was inoculated in LB broth and incubated for 20–72 h, at 27°C, and 160 rpm in a microbiological incubator; then bacterial stocks in 25% glycerol were prepared and conserved at −80°C. A total of 72 isolates were successfully grown and conserved in glycerol stocks.

### Isolate Identification

Partial PCR amplification of the 16S rRNA gene was performed directly from the glycerol stocks, using the universal primers F799 (5′-AACMGGATTAGATACCCKG-3′), and 1492R (5′-AAGGAGGTGATCCANCCRCA-3′) (Hogg and Lehane, 1999; Chelius and Triplett, 2001). The PCR mix contained: 1 μL of 10 μM F799 primer, 1 μL of 10 μM 1492R primer, 2.5 μL of bacterial glycerol stock, and 10.5 μL Premix Ex Taq RR003A. The PCR was performed in a iCycler iQ thermocycler, with the following protocol: an initial denaturation step at 98°C for 1 min; 30 PCR cycles at 98°C for 10 s, 57°C for 30 s, 72°C for 1 min; and a final extension at 72°C for 5 min. PCR products were purified with the NucleoSpin Gel and PCR Clean-up kit from Macherey-Nagel, following the manufacturer guidelines.

DNA concentration in each purified PCR product was measured in a Nanodrop ND-1000 spectrophotometer. Capillary sequencing was carried out by CIMA Lab Diagnostics. Sequencing reads were searched against RDP SeqMatch (Cole et al., 2014) and BLASTn databases using default parameters. For the majority of the isolates, both databases provided the same taxonomical assignment. If not, BLASTn^3^ taxonomical assignment prevailed. The reference sequences selected belonged to GenBank 16S partial sequences and were used for building the phylogenetic tree. The reference sequences and the sequences of the isolates were aligned by Clustal Omega web service^4^ (Madeira et al., 2019) with default parameters except for the number of combined iterations, max guide tree iterations, and max HMM iterations, that were shifted from default to five in all of them. The alignment tree distances resulting from Clustal Omega were used as basic data to create the circular cladogram tree in iTOL^5^ (Letunic and Bork, 2019).

### Bacterial Endophytes Characterization

#### Growth on Nitrogen-Free Medium

The endophyte isolates were tested for their capability to fix or scavenge nitrogen using NFC medium (10 g/L mannitol, 0.2 g/L MgSO_4_.7 H_2_O, 0.2 g/L KH_2_PO_4_, 0.2 g/L NaCl, 0.2 g/L CaSO_4_.2 H_2_O, 5 g/L CaCO_3_, 15 g/L European bacteriological agar; pH 7.2), based in Ashby’s mannitol agar (Liu et al., 2016; Li et al., 2018). Microorganisms were picked from the glycerol stocks (10 μL), streaked on NFC medium, and incubated at 30°C for 7 days. Those plates with positive growth were re-streaked over fresh NFC plates twice (each 7 days). Only the plates with consistent bacterial growth after 21 days were considered positive isolates for nitrogen-free medium growth trait. An *Azotobacter vinelandii* DSMZ 85 strain was used as a positive control microorganism.

#### Inorganic Phosphate Solubilization

For the detection of mineral phosphate solubilizer microorganisms, NBRIP agar was used as the culture medium: 10 g/L glucose, 5 g/L Ca_3_(PO_4_)_2_, 5 g/L MgCl_2_⋅6 H_2_O, 0.25 g/L MgSO_4_⋅7 H_2_O, 0.2 g/L KCl, 0.1 g/L (NH4)_2_SO_4_, 15 g/L European bacteriological agar, pH 7–7.2 (Nautiyal, 1999; Truyens et al., 2013). Glucose was dissolved in a small volume of sterilized dH_2_O, filter-sterilized (0.45 μm), and then added to the sterilized medium.

Each isolate was tested in two different plates, placing in each of them five 10 μL-drops from the corresponding bacterial glycerol stock. After 7 days at 27°C in darkness, clear halos around positive isolates were measured. These isolates were classified as fast solubilizers. Seven days later, solubilization halos were measured again, and those isolates with new clear halos were classified as slow solubilizers. The IPS ratio (Inorganic Phosphate Solubilization ratio) between the halo diameter and the colony diameter was also used as a classification parameter (Batista et al., 2018). A *Bacillus megaterium* var. *phosphaticum* DSMZ 3228 strain was used as a positive control for inorganic phosphate solubilization.

#### Siderophore Production

Isolates were tested as siderophore producers with the CAS-agar protocol developed by Schwyn and Neilands (1987) and modified by Cordero et al. (2012). Firstly, all the PYREX glasswares were deferrated, rinsing with 10% HCl (vol/vol) overnight and five consecutive washes with dH2O. Then, the CAS-Fe-HDTMA dye was prepared (1 L): 10 mL FeCl_3_ 10 mM dissolved previously in 100 mM HCl, 590 mL 1 mM Chrome azurol sulfonate, and 400 mL 2 mM HDTMA. The solution was autoclaved (25 min, 121°C) in an opaque PYREX bottle and stored at room temperature. After that, the CAS-agar was prepared, containing 30.24 g PIPES, 1 g/L NH_4_Cl, 3 g/L KH_2_PO_4_, 20 g/L NaCl, adjusting at a final pH of 6.8, and finally adding 9 g/L agar noble. After autoclaving (20 min, 121°C), 30 mL of filter-sterilized (0.45 μm) 10% (w/v) casamino acids, 10 mL of filter sterilized (0.45 μm) 20% glucose (w/v) and 100 mL of previously prepared CAS-Fe-HDTMA solution were added to the medium and dispensed in plates. Each isolate was tested in the same way as in the phosphate solubilization assay. After 7 days at 27°C in darkness, yellow-orange halos around positive isolates were measured. These isolates were classified as siderophore producers. The MCI ratio (Metal Chelation Index ratio) between the halo diameter and the colony diameter was also used as a parameter to evaluate the siderophore production (Batista et al., 2018). A *Pseudomonas* sp. DSMZ 25842 strain was used as a positive control for siderophore production.

#### Plant Hormones Production

The production of plant hormones by bacterial isolates was tested by growing each isolate in 5 mL of LB broth supplemented with filter-sterilized (0.45 μm) 5 mM L-Tryptophan, for IAA production (Lin et al., 2015; Gilbert et al., 2018). Isolates were grown in triplicates for 20 h at 28°C with 250 rpm shaking in 50 mL sterile centrifuge tubes. OD at 600 nm of all isolates was measured, but those that did not reach a minimum OD_600_ value of 0.6 were not considered for hormone concentration measurements. In resume, only 55 isolates were considered for hormone production analyses. The cultures were centrifuged at 5,200 rpm for 10 min, and supernatants were transferred to clean 12 mL tubes and stored at −80°C until hormone quantification. A *Pseudomonas* sp. DSMZ 25842 strain was used as a previously known IAA producer. Final concentration for each replicate was calculated after subtracting the control (LB medium) hormonal concentration and dividing by the OD_600_ value.

The content of acidic hormones (IAA; jasmonic acid, JA; jasmonoyl isoleucine, JA-Ile; abscisic acid, ABA; and salicylic acid, SA) and CKs (isopentenyladenine, iP; isopentenyladenosine, iPR; trans- and cis-zeatin, tZ and cZ; trans- and cis-zeatin riboside, tZR and cZR; dihydrozeatin, DZ; dihydrozeatin riboside, DZ) were analyzed by high performance liquid chromatography-electrospray-high-resolution accurate mass spectrometry (UHPLC-ESI-HRMS).

The procedures for the determination of acidic hormones and CKs are different and were performed separately using two different aliquots from the same sample/culture. The quantification was carried out in a Dionex Ultimate 3000 UHPLC device coupled to a Q Exactive Focus Mass Spectrometer (Thermo Fisher Scientific), equipped with an ESI source, a quadrupole mass filter, a C-Trap, a HCD collision cell, and an Orbitrap mass analyzer, following the methodology elaborately described in Silva-Navas et al. (2019).

The content of IAA, JA, JA-Ile, ABA, and SA was analyzed as follows: for each triplicate of every bacterial culture, and for three replicates of the pure culture medium (without bacteria, as a blank), aliquots of 90 μL of culture broth were added to 10 μL of internal standard (1000 ng ml^–1^ of deuterium-labeled internal standards in metanol), 150 μL of MeOH, and 150 μL of acetic acid 0.133%, and centrifuged at 20,000 *g* (Sigma 4–16 K Centrifuge) for 10 min before the injection in the UHPLC-ESI-HRMS system, using exactly the same conditions detailed in Silva-Navas et al. (2019), and the same identification and quantification procedure.

For the analysis of the production of CKs by endophytic bacterial isolates, 100 μL of the culture medium for each bacteria and replicate were mixed with 25 μL of internal standard (100 ng/mL of each standard in methanol), 225 μL of methanol and 150 μL of formic acid 0.04% and centrifuged at 20.000 *g* for 10 min before the injection of the sample. Three aliquots of 100 μL of the pure culture medium without bacteria were subjected to the same procedure, with the purpose of serving as a blank for the determination of the concentration of hormones produced by the bacteria. The measurement conditions, detection, and quantification have already been described in Silva-Navas et al. (2019).

### Statistical Analysis

For comparison between hormonal productions by genus, ANOVA signification tests were carried out followed by HSD Tukey *post-hoc* tests. The statistical tests were performed with the stats package in R (RStudio Team, 2016). The *p* ≤ 0.05 was used as statistically significant threshold.

## Results

### Taxonomic Diversity of SHA Pre-treated Cucumber Culturable Endophytic Microbiota

The number of viable endophytic bacterial isolates obtained from three different plants of cucumber previously grown in the presence of 100 ppm C SHA in the nutrient solution was 72. For the taxonomic identification of the isolates, BLASTn and RDP SeqMatch databases were utilized. Both identifications were similar, with only a misleading identification in one isolate (CR329.a, Supplementary Table S1). Most of the microorganisms identified (97% of the isolates) showed ≥ 97% of similarity with reference sequences in BLASTn database.

**FIGURE 1:**
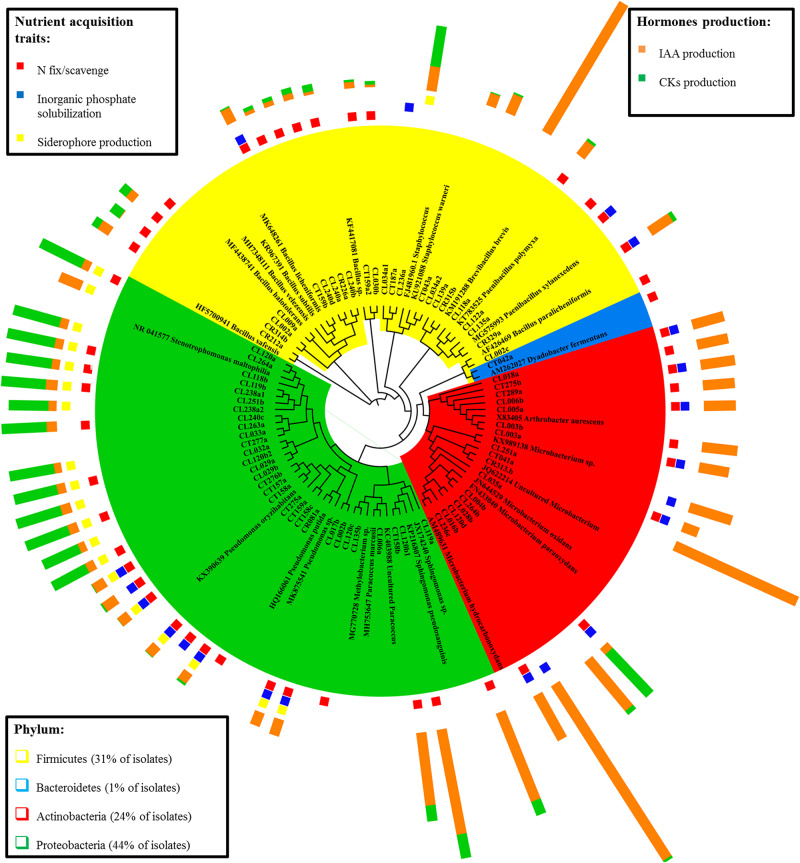
Cladogram representing the identification of the endophytic bacterial isolates and the corresponding plant growth promotion traits. Isolates are grouped by their sequence similarity to the BLASTn reference sequence. Phylum taxonomic level is showed by color: *Firmicutes* (yellow), *Bacteroidetes* (blue), *Actinobacteria* (red), and *Proteobacteria* (green). Plant growth promotion traits are separated by nutrient acquisition traits (presence or absence) and hormone production.

The cladogram tree represents the closest classification of isolates to reference sequences in BLAST (Figure 1). Most of the isolates belonged to the phylum *Proteobacteria* (44%), followed by *Actinobacteria* (24%), *Firmicutes* (31%), and *Bacteroidetes* (1%) (class, order, and family distributions of isolates are shown in Supplementary Figure S1). *Proteobacteria* isolates were the most diverse group, with five different families (*Xanthomonadaceae*, *Pseudomonadaceae*, *Sphingomonadaceae*, *Rhodobacteraceae*, and *Methilobacteraceae*) representing two different classes (*α-* and *γ-proteobacteria*). Both *Actinobacteria* and *Firmicutes* were represented by only one phylogenetic class: *Actinobacteriia* and *Bacilli*, respectively. In each class, bacteria from two (*Micrococcaceae* and *Microbacteriaceae*) and three (*Staphylococcaceae*, *Paenibacillaceae*, and *Bacillaceae*) different families were identified. *Bacteroidetes* phylum had only one isolate belonging to *Cytophagaceae* family. The most represented isolated species was *Stenetrophomonas maltophilia* (15 isolates), followed by *Arthrobacter* aurescens (seven strains) and *Pseudomonas oryzihabitans* (seven strains). The cladogram confirmed the identification by BLASTn and RDP SeqMatch.

### Plant Growth-Promotion Traits in Endophyte Isolates

Endophytic isolates were screened for their *in vitro* plant growth-promoting traits (PGP). The traits selected were those related to the mineral nutrient acquisition (nitrogen fixation/scavenging, inorganic phosphate solubilization, and siderophore production) and those associated with plant growth regulators (IAA and CKs plant hormones) production. Isolates clustering together (Figure 1) showed similar PGP performance according to the studied traits, but the functional strain diversity was highlighted as well.

**FIGURE 2:**
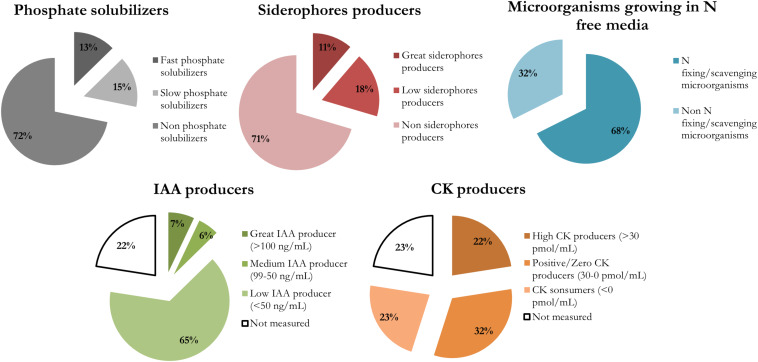
Prevalence of each plant growth promotion trait in the endophytes isolated from cucumber plants pre-treated with a sedimentary humic acid. The traits evaluated were: inorganic phosphate solubilization at 14 days, siderophore production at 7 days, capability of growing in a N-free medium, and phytohormones production (IAA and CKs).

The biological nitrogen fixation/scavenging was the most prevalent PGP trait within the nutrient acquisition features studied, with 68% of isolates being able to grow in an N free medium (Figure 2). This trait is showed by diverse phylogenetic groups (Figure 1).

Regarding inorganic phosphate solubilization, it was performed by only 28% of isolates. They were classified as: fast solubilizers, which solubilized phosphate after 7 days and had the greatest IPS ratios after 14 days; or slow solubilizers, whose halos of solubilization were visible not at 7 days but after 14 days, or showed small IPS ratios. All the fast solubilizers (9 isolates) were identified as *Pseudomonas* genus. The slow phosphate solubilizers were more diverse, and there were 11 isolates from six different genera: *Arthrobacter*, *Paenibacillus*, *Microbacterium*, *Stenotrophomonas*, and *Staphylococcus* (Supplementary Table S2).

Most of the isolates producing siderophores able to chelate iron (29% of isolates) belong to *Pseudomonas* and *Stenetrophomonas* genera (Figure 2 and Supplementary Table S3). Those isolates producing a MCI ratio higher than 1.5 were classified as great siderophore producers, all the identified *Pseudomonas* strains pertained to that group.

Although the production of a wide group of plant hormones (listed section “Materials and Methods”) has been tested, only IAA, cZ, cZR, iP, and iPR were detected in our cultured endophytic isolates.

**FIGURE 3:**
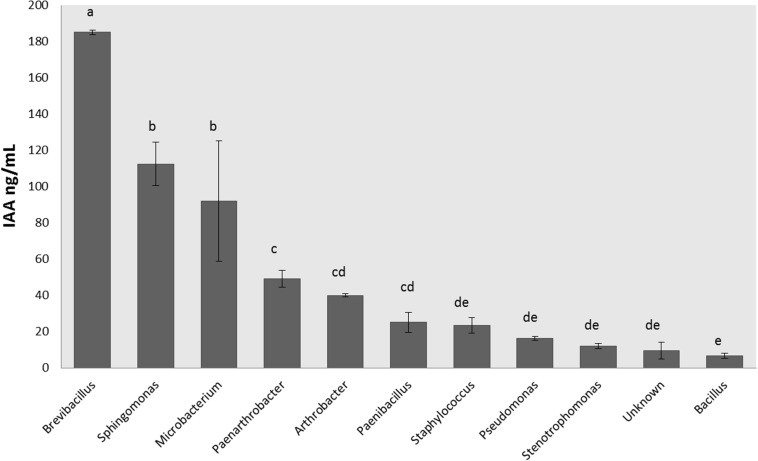
Production of indole-3-acetic acid by endophytic isolates according the genus. Indole-3-acetic acid production was measured in ng per mL after 20 h of growth at 28°C in LB medium supplemented with Trp 5 mM. Production was measured in triplicates for each isolate, and the final concentration was obtained after standardization. Bar errors represent the standard error. Letters represent the significant groups after ANOVA and HSD Tukey *post-hoc* tests. Signification threshold: *p* ≤ 0.05.

There were 16 isolates that did not grow appropriately in LB broth supplemented with 5 mM Trp, so the hormonal production was not measured for those bacteria (Supplementary Table S1). The rest of the isolates were able to produce IAA and CKs. IAA production ranged between 1 and 245 ng/mL (Figure 3), and the isolates were classified according to their IAA production in three levels: low producers (<50 ng/mL), medium producers (50–99 ng/mL) and great producers (>100 ng/mL). Most of the isolates were low producers (65% of all isolates), but 7% of isolates (5 strains) were great IAA producers and identified by BLASTn as *Microbacterium paraoxydans*, *Sphingomonas pseudosanguinis*, *Sphingomonas* sp., an uncultured *Microbacterium*, and *Brevibacillus brevis*.

**FIGURE 4:**
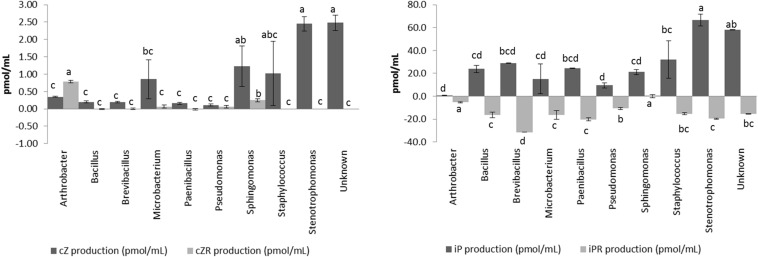
Production of cytokinins (cZ, cZR, iP, and iPR) by endophytic isolates according the genus. Cytokinin production was measured in pmol per mL after 20 h of growth at 28°C in LB medium supplemented with 5 mM L-Tryptophan. Production was measured in triplicates for each isolate, and the final concentration was obtained after standardization. Bar errors represents the standard error. Letters represents the significant groups after ANOVA and HSD Tukey *post-hoc* tests. Signification threshold: *p* ≤ 0.05.

The four different CKs detected (cZ, cZR, iP, and iPR) showed different dynamics in the isolates (Figure 4) and we have classified the CKs producers according to net CKs production. This net production was categorized as highly positive (>30 pmol/mL, CKs great producers), positive or zero (0–30 pmol/mL, CKs low or no producers), or negative (<0 pmol/mL, CKs consumers). Most of the isolates consumed part of the iPR initially present in the culture broth, and some of them produced larger amounts of iP (Figure 4). On the other hand, with the exception of *Arthrobacter*, all isolates produced smaller quantities of cZR than cZ. CKs net production appears as a diverse and essential trait for different endophytic bacterial taxa. There were 16 isolates with a high net production, ranging between 45 and 72 pmol/mL of total CKs. The most represented genera among these CKs producers were *Stenotrophomonas maltophilia* (14 isolates) strains (Supplementary Table S1). In general, high CKs net production was accompanied by low levels of IAA production.

## Discussion

### Plant Growth Promotion Traits of Cultured Endophyte Taxa From SHA-Treated Plants

Endophytic communities are commonly shaped by the soil, being the roots the main entrance door (Hardoim et al., 2008, 2015; Reinhold-Hurek et al., 2015). Another important source of bacterial endophytes is the vertical inheritance of endophytes through the seeds (Johnston-Monje and Raizada, 2011; Hardoim et al., 2012; Truyens et al., 2013, 2015; Khalaf and Raizada, 2016, 2018; Nelson, 2018). These vertically transmitted endophytes have been specially selected by evolutionary forces; therefore conforming an interesting option for applications in agriculture. In our experimental design, in which cucumber plants have grown in hydroponics, presumably most of the culturable endophytes isolated are inherited from the seeds of *Cucumis sativus* var. *Ashley*.

Despite only being a fraction of the total number of bacteria living within the plant, the distribution of the cultured endophytes among different phyla (*Proteobacteria*, *Actinobacteria*, *Firmicutes*, and *Bacteroidetes*) was similar to the predominant composition of bacterial communities in rhizospheres and endospheres of angiosperms plants (Hardoim et al., 2015), such as barley (Bulgarelli et al., 2015), rice (Hameed et al., 2015), wheat (Liu et al., 2017), the plant model *Arabidopsis thaliana* (Lundberg et al., 2012), and other plants (Fitzpatrick et al., 2018).

In this work, PGP traits related to the improvement of nutrient acquisition or to the production of plant growth hormonal regulators have been assessed. In the case of the nutrition-related traits, most of the isolates (68%) were able to grow in N-free medium. This trait implies the potential capability of these microorganisms to fix atmospheric nitrogen or the scavenging of trace N-compounds from the atmosphere (such as NH_3_ or N_2_O) (Zuluaga et al., 2020). Juraeva et al. (2006) already reported a correlation of N content and endophytic nitrogen fixation in cucumber plants, especially in roots, based on the analysis of nifH gene copy numbers, indicating the relevance of this trait in cucumber plants, although further assays should be carried out to confirm the actual biological nitrogen fixation or scavenging activity of our isolates.

Siderophore production and phosphate solubilization were the less common traits in our isolates. Bacterial siderophore iron complexes can contribute to iron uptake in plants, and they might confer a certain level plant defense induction through limiting the availability of Fe for pathogens (Ahmed and Holmström, 2014). Phosphate solubilization is an ecologically important trait because P is the second major limiting nutrient for plant growth despite its abundance in soils (Weyens et al., 2009; Khalaf and Raizada, 2016). Phosphorus is habitually found in non-plant-available forms, such as tricalcium phosphate or phytate, so microbial solubilization of inorganic phosphate or mineralization of organic phosphorus would enhance its bioavailability for plants (Mahanty et al., 2016). Within our endophytic isolates, only *Pseudomonas* sp. showed both siderophore production and phosphate solubilization traits, producing the greatest MCI and IPS ratios (Supplementary Tables S2, S3). *Pseudomonas* genus is a well-known siderophore producer, especially by the synthesis of pyoverdine (Kloepper et al., 1980; Gamalero and Glick, 2011), as well as an inorganic P solulibilizer (Browne et al., 2009). *Stenotrophomonas maltophilia* isolates were good siderophore producers (Egamberdieva et al., 2016; Singh and Jha, 2017), while *Arthrobacter* strains were promising inorganic phosphate solubilizers.

On the other hand, the plant growth hormonal regulators produced by the isolated bacterial endophytes were indole-3-acetic acid (IAA) and several cytokinins (cZ, cZR, iP, an iPR). Bacterial production of IAA has been extensively studied (Dodd et al., 2010; Hameed et al., 2015; Fidalgo et al., 2016; Gilbert et al., 2018; López et al., 2018; Afzal et al., 2019; Zuluaga et al., 2020) but, in contrast with most of the works previously referenced, in our study the IAA production has been analyzed by means of UHPLC-ESI-HRMS. This technique has lower detection limits than the Salkowski reagent method (Gordon and Weber, 1951).

The increment in IAA plant concentration promotes the cell proliferation, enlarges the root system, increases the root biomass, changes the root architecture, and enhances nutrient and water uptake efficiency (Dodd et al., 2010; Egamberdieva et al., 2016; Gilbert et al., 2018). The highest production of these plant hormones in our isolates was found in *Brevibacillus*, *Microbacterium*, and *Sphingomonas* genera (Figure 3). The bacterial production of plant-like growth regulators has been commonly associated with host-microbe cross-talk and plant colonization (Spaepen et al., 2007; Spaepen and Vanderleyden, 2011; Carvalho et al., 2014; Koul et al., 2015). Defez et al. (2017) also reported that IAA-overproducing transformed endophytic diazotrophs improved their nitrogen-fixing capacity both *in vitro* and in inoculated rice-roots.

The production of cytokinins by bacteria, although widely known, is rarely taken into account in PGP bacterial characterization, and it only has been measured in a few works (Timmusk et al., 1999; Dodd et al., 2010 and references therein). Our results showed that the isolated endophytic bacteria produced mainly iP, and also small amounts of cZ, while iPR was transformed/consumed from the medium. The high production of iP by some of these bacterial isolates could have important effects on plant development since iP is considered one of the most active CKs in the plant (Osugi and Sakakibara, 2015). In general, the action of cytokinins in plants is related to the formation of shoots, chloroplastic maturation, cell expansion, stomatic conductance, and meristematic tissue differentiation (Cassán et al., 2014; Osugi and Sakakibara, 2015). Recently, new effects have been found for this family of hormones, such as the role of cZ in biotic and abiotic response, or nutritional status (Großkinsky et al., 2013, 2016; Schäfer et al., 2015; Silva-Navas et al., 2019).

### Relationships Between the PGP Traits of Culturable Endophytes in SHA-Treated Plants and the Mechanism of Action of SHA in Plants

Mora et al. (2010) reported that SHA is able to promote nitrate root uptake and nitrate reductase activity in cucumber plants. Other studies showed that humic acids extracted from different sources were able to induce the expression of plant genes directly involved in nitrate transport and further assimilation, as well as to enhance the root H^+^-ATPase activity (Trevisan et al., 2010; Jannin et al., 2012; Olaetxea et al., 2018). But the detailed mechanisms through which HS enhance the uptake of different nutrients are still unveiled and, with the exception of the work by de Hita et al. (2018), there are no studies exploring the effects of HS on endophytic microbiomes. It might be possible that the thriving of certain endophytic bacteria in roots could lead to an acidification of the external pH, which could concomitantly contribute to an enhanced assimilation or bioavailability of several nutrients (i.e., nitrate or Fe). Therefore, the effects observed upon the treatment with HS could be additional to (or mediated by) the effects corresponding to bacterial endophytes.

Other studies have shown that HA-metal-phosphate complexes led to an increase in the internal utilization of P by plants (Urrutia et al., 2014; Jindo et al., 2016), with higher concentrations of soluble phosphate in plant tissues. The fact that several families of endophytes isolated from SHA pre-treated plants were able to mobilize inorganic P is also compatible with a possible P solubilization from those fractions precipitated with Fe or Ca in the apoplast (Snowden and Wheeler, 1995). Therefore, both SHA and endophytes could contribute to the mobilization of internal fractions of precipitated P.

A similar reasoning might be applied to Fe plant nutrition. Various studies have shown the ability of HS to improve Fe root uptake and further assimilation in cucumber (Pinton et al., 1999; Aguirre et al., 2009; Zanin et al., 2019), with a significant activation of Fe-deficiency root responses even under Fe sufficient conditions (Aguirre et al., 2009), and an increase in the physiologically active Fe fraction (1N HCl-extractable Fe, related to the chlorophyll content). In this framework, those endophytes producing siderophores could also contribute to this process through the solubilization of Fe precipitated in apoplast. Thus, as in this case of P, the effects observed upon SHA treatment improving Fe plant nutrition are compatible with a positive action of specific endophytic groups.

This hypothetical synergistic action of SHA and endophytic microbiota in the whole mechanism responsible for the growth enhancing effect of SHA in cucumber plants could also be extended to the case of plant hormone action. Several studies have reported that the shoot- and root-growth promoting action of SHA in cucumber is regulated by IAA, ABA, and some families of CKs, principally trans-zeatin (tZ) and adenine-based CKs (Mora et al., 2010; Olaetxea et al., 2015, 2018). Most of the endophytic bacteria isolated from SHA pre-treated cucumber plants were able to produce significant amounts of IAA abd CKs (Figures 3, 4), what is also compatible with a potential cooperation between the biochemical action of SHA and bacterial endophytic activity in plant tissues. Regarding CKs, the cultured endophytes promoted the synthesis of cZ and not tZ, which is the main CK involved in the SHA shoot growth promoting effect. However, a recent study has shown that the cZ:tZ ratio plays a very relevant role in the regulation of plant responses to P deficiency (Silva-Navas et al., 2019). It could therefore be possible that the ability of endophytes to produce cZ has some influence in the improvement of the adaptation of SHA-treated cucumber plants to low concentrations of available P in the nutrient solution.

## Conclusion

Endophytic microorganisms with PGP traits are a promising tool to improve the crop production due to its natural presence in plants tissues, which confer them an ecological advantage against rhizosphere microorganisms. This study also highlights the importance of seed microbiome, as the bacterial endophytes have been isolated from cucumber plants grown in a hydroponic system with a minimized entrance of microorganisms, compared to the size and variety of microbial communities present in soils.

The cultivable endophytes isolated from plants treated with a SHA present a relevant capacity to affect some processes related to plant mineral nutrition and hormonal signaling pathways. In addition to that, all these plant growth promotion traits can be evolved in a complementary, additive or synergistic way with the main mechanisms activated upon SHA application. One of the perspectives to explore in depth in future works would be the actual PGP activity of these isolated endophytic bacteria, applied either alone, as a consortium, or using SHA as a carrier.

## Data Availability Statement

All datasets generated in this study are included in the article and the Supplementary Material. Isolate sequencing reads were deposited in GenBank under accession numbers MN512151 to MN512214.

## Author Contributions

DD, MF, and JG-M conceptualized and designed the study and carried out the data analysis. DD and YR performed the experimental work. AZ assessed the hormone detection. DD did statistical analysis. DD, MF, JG-M, and AZ prepared the manuscript. All authors contributed to the article and approved the submitted version.

## Conflict of Interest

The authors declare that the research was conducted in the absence of any commercial or financial relationships that could be construed as a potential conflict of interest.

## Footnotes

1 http://www.fao.org/fileadmin/templates/wsfs/docs/Issues_papers/HLEF2050_Global_Agriculture.pdf

2 http://www.fao.org/faostat/en/3data

3 https://blast.ncbi.nih.gov/

4 https://www.ebi.ac.uk/Tools/msa/clattalo

5 Interactive Tree of Life, https://itol.embl.de/

## Supplementary Material

FIGURE S1 Isolates distribution by phylum, class, order, and family.

TABLE S1 Identification and PGP characterization of endophytes extracted from SHA pre-treated cucumber plants. The identification used as reference databases the BLASTn and RDP Seq-Match assignment of 16S rRNA sequences.

TABLE S2 Inorganic phosphate solubilization (IPS) ratios for those isolates able to solubilize the inorganic phosphate after 7 or 14 days after incubation.

TABLE S3 Metallic chelation index (MCI) ratios for those isolates producing siderophores after 7 days of incubation.

FILE S1 16S partial sequences of the isolates.
