# Supplementary figures and images for: Culturable Bacterial Endophytes From Sedimentary Humic Acid-Treated Plants
Source: Front Plant Sci. 2020 Jun 19;11:837. doi: 10.3389/fpls.2020.00837 (PMC7316998; doi:10.3389/fpls.2020.00837)

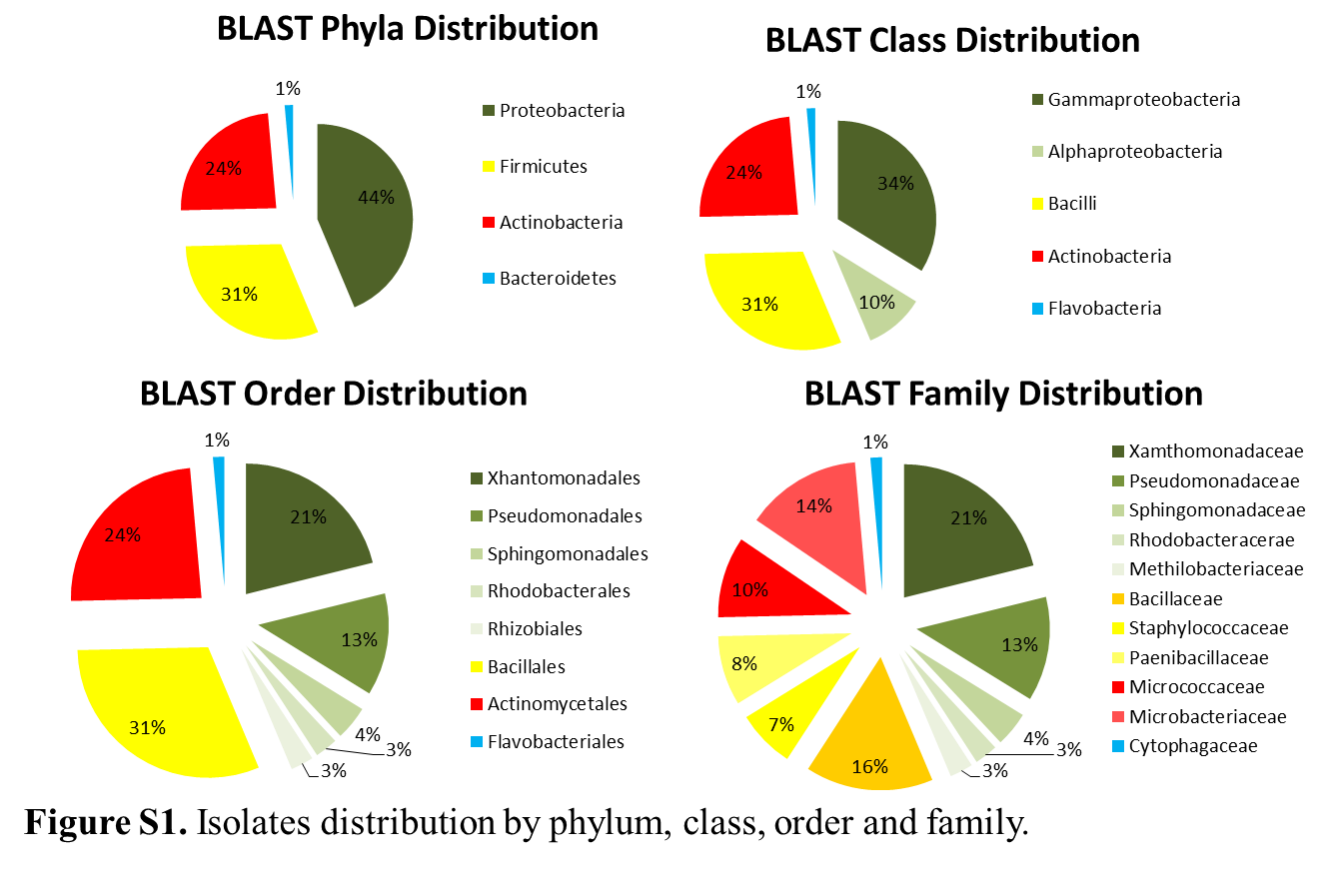

Supplement: FIGURE S1 — Isolates distribution by phylum, class, order, and family. [file Image_1.TIF]
